# Does “a picture is worth 1000 words” apply to iconic Chinese words? Relationship of Chinese words and pictures

**DOI:** 10.1038/s41598-018-25885-9

**Published:** 2018-05-29

**Authors:** Shih-Yu Lo, Su-Ling Yeh

**Affiliations:** 1 Institute of Communication Studies, National Chiao Tung University, Hsinchu, Taiwan; 2 Center for General Education, National Chiao Tung University, Hsinchu, Taiwan; 3 Department of Psychology, National Taiwan University, Taipei, Taiwan; 4 Graduate Institute of Brain and Mind Sciences, National Taiwan University, Taipei, Taiwan; 5 Neurobiology and Cognitive Science Center, National Taiwan University, Taipei, Taiwan; 6 Center for Artificial Intelligence and Advanced Robotics, National Taiwan University, Taipei, Taiwan

## Abstract

The meaning of a picture can be extracted rapidly, but the form-to-meaning relationship is less obvious for printed words. In contrast to English words that follow grapheme-to-phoneme correspondence rule, the iconic nature of Chinese words might predispose them to activate their semantic representations more directly from their orthographies. By using the paradigm of repetition blindness (RB) that taps into the early level of word processing, we examined whether Chinese words activate their semantic representations as directly as pictures do. RB refers to the failure to detect the second occurrence of an item when it is presented twice in temporal proximity. Previous studies showed RB for semantically related pictures, suggesting that pictures activate their semantic representations directly from their shapes and thus two semantically related pictures are represented as repeated. However, this does not apply to English words since no RB was found for English synonyms. In this study, we replicated the semantic RB effect for pictures, and further showed the absence of semantic RB for Chinese synonyms. Based on our findings, it is suggested that Chinese words are processed like English words, which do not activate their semantic representations as directly as pictures do.

## Introduction

Printed words serve two functions: to convey sounds as well as meanings from their symbolic forms. For words in an alphabetic writing system, the orthography-phonology mapping is fairly consistent. For example, the spelling-phonology consistency for monosyllabic English words is 70%^1^. In contrast, the orthography-meaning mapping in an alphabetic writing system is arbitrary (e.g., park, dark, part, pork all look alike but they are not semantically similar). However, in an ideographic writing system such as Chinese, no grapheme-to-phoneme correspondence rule exists, whereas the orthography-meaning mapping is more reliable; for example, Chinese words with enclosed structures like  all indicate something being surrounded. This reliable orthography-meaning correspondence of Chinese words resembles the reliable form-to-meaning correspondence of pictures (e.g., if a picture contains the image of a leg, it normally indicates something animate). Indeed, when Americans were asked to recall Chinese characters they just saw, they tended to draw pictures like people and shelf and described that Chinese characters were like pictures^2^. Based on the systematic orthography-meaning mapping in Chinese and the prevalent view of Chinese characters by non-readers, a reasonable conjecture is that the meaning of a Chinese word is accessed from its form more directly than that for English. This is what we aim to examine in this study; specifically, we adopted a novel approach by using the repetition blindness paradigm to test whether Chinese characters are processed like pictures.

## Nature of Chinese characters

Chinese characters are the basic writing units of Chinese script. A single character can be a word by itself, although it also can be combined with other characters to form a compound word. Hereafter we use “word” to indicate one-character words, unless otherwise stated. Each word corresponds to one morpheme and one syllable, and as a result, written Chinese has generally been considered a morphosyllabic system^3^. These characteristics of Chinese words are rooted in a writing system in which meaning is carried by pictographic or ideographic representations. As is well known, the original Chinese characters were created through processes that largely depended on picture-like properties. For example, the word  (meaning “mountain”) is a pictograph that looks like a mountain. This pictographic quality can be beneficial for a large population of Chinese readers who speak different dialects. However, during the development and evolution of Chinese script, most Chinese words have become less pictographic. For example, the word  (”horse”) no longer looks like a horse, even though it nevertheless generates a vivid impression of one in some Chinese readers^4^.

## Positive evidence for picture-like processing of Chinese words

A series of studies have attempted to examine whether Chinese words are pictographic^5–8^. For example, Luk and Bialystok^5^ asked a group of participants, who did not have any prior knowledge about the Chinese language, to guess the meanings of Chinese words from their orthographies, in a task where they had to pick the answer from two options. For a subset of Chinese words, called “iconic characters”—one-character words that resemble the objects they refer to—the guessing accuracy was above chance (a mean of 7.83 out of 10 characters). A consistent result of Chinese characters being picture-like was also found in an earlier study conducted on a group of Israeli teenagers and adults who did not have any knowledge about the Chinese language^8^.

For pictures, our visual system can extract the meanings instantly^9^. Given Chinese words are more iconic than their alphabetic counterparts, a reasonable conjecture is that they can activate the meanings more directly. Indeed, the issue whether the closer orthography-meaning relationship in Chinese exerts any differential effect in processing has long been debated^3,10–17^. Some studies support a closer orthography-meaning relationship for Chinese words than for English words, such as a more pronounced Stroop effect caused by Chinese words than for English words^10^. The distinct mechanisms for Chinese word and English word processing are also manifested by a recent neurophysiological study^18^; the finding that a later ERP signal difference was found between Chinese words and pictures for Chinese readers than that between English words and pictures for English readers suggests that Chinese words and picture processing share common mechanisms, as manifested by their common early ERP components. A recent fMRI study^19^ also offers evidence for a common processing module but differential activation patterns for Chinese and English words. Native English and Chinese readers were presented with Chinese words, English words, and objects. The visual word form area (VWFA) was activated when subjects viewed words of their native language (Chinese words for Chinese readers, and English words for English readers). Interestingly, English readers showed differential amounts of signal change for English words and objects, while Chinese readers showed equal amounts of signal change for Chinese words and objects. The similarity in brain activities for Chinese words and objects implies that Chinese words may be processed more like pictures.

## Negative evidence for picture-like processing of Chinese words

There is also conflicting evidence suggesting that Chinese words are processed just like English words, without a particularly close orthography-meaning relationship. For example, Liu^13^ presented a prime (e.g., , “*water*”, for a Chinese target, or *water* for an English target) for two seconds prior to the presentation of the target. The participants were asked to judge whether a given Chinese word (e.g., ,”*river*”, which is semantically related to “water”) or an English word (e.g., *river*) is semantically associated with the prime. The results were similar, regardless of whether Chinese or English words were used. They also used pictures as the targets, and found faster responses than targets of Chinese words. These results led Liu^13^ to conclude that Chinese words are no more picture-like than English words.

Recent fMRI studies also showed that the cortical regions involved in Chinese word processing overlap more with those for English word processing than for picture processing^20,21^. These studies are consistent with a view that word recognition is a process independent of object recognition. Although English and Chinese words are physically different, they are both linguistic materials that should involve the same cortical area. A candidate area for word-specific processing is the left occipitotemporal cortex (OTC), which responds to both English and Chinese words^22^. A cortical area that has been of particular interest termed VWFA, as mentioned in the previous section, is located in a specific region of the left OTC, and has been shown to respond more to words than other objects^23–26^.

## Possible reason for the discrepancy

As mentioned above, Chinese words were shown to be more picture-like in some studies but not in others. The discrepancy may be due to multiple levels of information processing for a long duration. For example, in Liu’s^13^ study, the prime-target interval was two seconds. In the fMRI studies, the Chinese characters were presented for a long period of time ranging from 800 ms^20^ to three seconds^21^. In contrast, the EEG study by Yum *et al*.^18^ that revealed a closer picture-word similarity for Chinese words than English words investigated a much earlier time window; the ERPs triggered by Chinese words overlapped with those triggered by pictures in a temporal window up to 300 ms after stimulus onset, while the ERPs for English words deviated from the ERPs for pictures as early as 150 ms. Why are Chinese words processed more like pictures in early stages of information processing flow? Possibly, both systems involve direct form-to-meaning activations, as opposed to the English system that may involve a less direct form-sound-meaning activation.

In order to test whether Chinese words and pictures both involve direct form-to-meaning activations in early stages of information processing, we chose the *repetition blindness* (RB) paradigm to further examine this issue. The RB paradigm measures participants’ identification accuracy of target items in a rapid serial visual presentation (RSVP). In addition, stimulus presentation duration in an RB task is usually less than 100 ms, and thus the effect is more sensitive to visual representations that are extracted at the early stages of information processing^27,28^.

## Repetition blindness

RB is indexed by lower accuracy when the second target item is identical, or similar, to the first target item. For example, RB occurs with two identical words^29^, pictures similar in shape but different in meaning such as a picture of a closed umbrella and a picture of a carrot^30^, pictures with the same identity but differing in orientation or size^30^, or a picture and a word that refer to the same object such as a picture of a *sun* and the word “*sun”*^31^.

According to a prominent theory of RB^29^, what happens in RB is that each item presented in RSVP first activates its corresponding *type* representation, followed by the construction of a *token* representation. *Type* refers to a pre-existing representation that is used for recognition (i.e., “what”) and *token* is a spatiotemporal representation (i.e., “where and when”) of that type. Building a token representation is a time-consuming process. If an item is presented twice in temporal proximity, the same *type* representation fails to be tokenized into separate token representations. Consequently, the observer can perceive “what” this item is, but cannot tell apart its first and the repeated appearances, because the “when” information has not been consolidated for this short duration.

Bavelier^31,32^ further extended this model and postulated that the token individuation process is a dynamic and continuous process during which information from orthography, phonology, or semantics of a word can be tokenized. The token individuation process is followed by the consolidation process and only a stabilized token can be encoded into episodic memory for response. According to this framework, processes at both stages (individuation and consolidation) can cause RB, and once one kind of information (e.g., orthography) has already built a stabilized token, the other information (e.g., semantics) of the same item that comes later will be less likely to influence the effect of RB.

Paradoxically, RB has been found for semantically related pictures, such as pictures of a *helicopter* and an *airplane*^30^ but not for semantically related English words, such as *sofa*-*couch*^33^, *take*-*took*^27^, and *helicopter*-*airplane*^30^. Bavelier^32^ hypothesized that differences in the speed of semantic processing for the task at hand may explain the differences in RB for words and for pictures. According to this hypothesis, a written word accesses its orthographical representation much more quickly, so words with different forms can activate different type representation even if they share the same meaning (synonyms), thus yielding little or no RB. A picture activates its semantic representation directly^34–37^, so two pictures with identical or related meanings activate the same type representation, leading to an RB effect.

## Rationale of the current study

In this study, semantically related pictures or Chinese words were used as the critical items (C1 and C2) in RSVP. If the processing of Chinese words is similar to that of pictures, where the semantic representations are accessed more directly than English words, then we should observe an RB effect for synonyms. Specific to the RB paradigm used here, as the orthography-meaning mapping in Chinese is more reliable than the mapping in English, it is possible that such reliability of this mapping in Chinese will lead to semantics being activated earlier for Chinese than for English words. Therefore, a semantic RB effect may be obtained as was found for pictures^30^.

## Results

### Picture experiment

**Figure 1:**
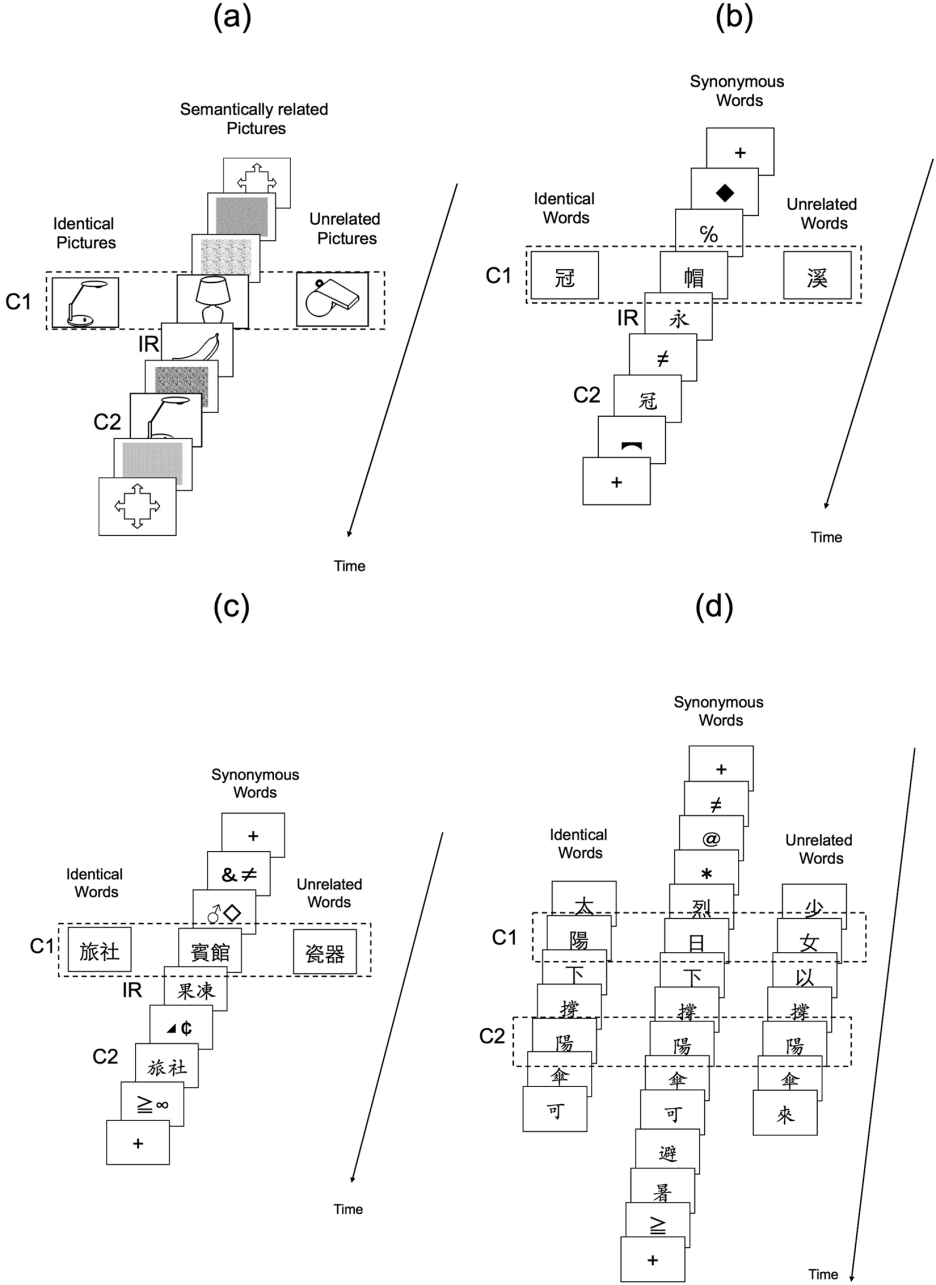
Schematic illustrations of the RSVP sequences in the (**a**) Picture experiment (pictures for the critical and irrelevant items were chosen from Wang’s^53^ study, and a written permission to use the object contour pictures shown here has been granted by *Chinese Journal of Psychology*) (**b**) Word-General experiment/one-character word condition (**c**) Word-General experiment/two-character word condition, and (**d**) Pictograph-Sentence experiment. IR stands for irrelevant item, C1 for the first critical item, and C2 for the second critical item. For each experiment, the relationship between C1 and C2 could be identical, semantically related (synonymous in some cases), or unrelated.

**Figure 2:**
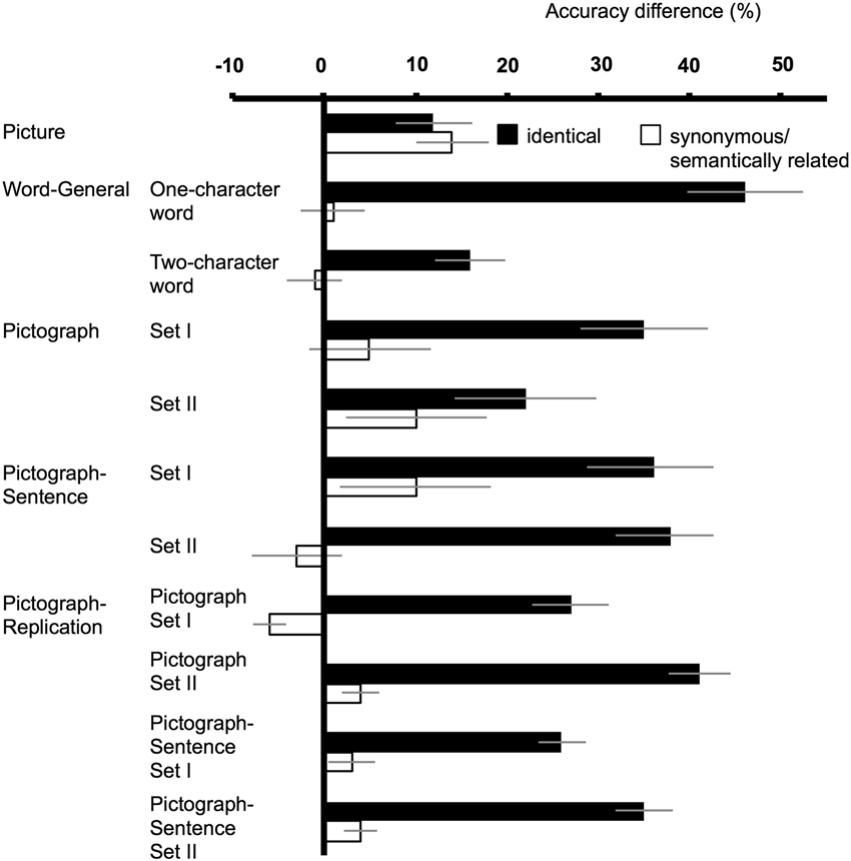
Magnitudes of RB (i.e., difference in accuracy between the denoted condition and the unrelated condition) across different experiments in this study. The error bars indicate one standard error of the mean across subjects.

We began with the *Picture* experiment (Fig. 1a), which aimed to replicate the finding that RB occurs for semantically related pictures^30^. Observers viewed a series of symbols and three pictures, with the first and the last of the pictures denoted as C1 and C2 (the first and the second critical items), and the middle one as IR (irrelevant item). The relationship between C1 and C2 could be identical, semantically related, or unrelated. In this and all subsequent experiments, accuracy (see Table 1 for the mean values) was measured by the proportion of trials where C1 and C2 were both reported correctly, regardless of their temporal order^29,33,38^, and were analyzed using logit mixed models^39^ in R. With the accuracy in the unrelated condition used as the baseline, the accuracy was significantly lower in the semantically related condition (*z* = −2.53, *p* = 0.01), demonstrating a semantic RB effect. In addition, a form repetition effect was also manifested, as the accuracy in the identical condition was significantly lower (*z* = −1.93, *p* = 0.05) than that in the unrelated condition (To illustrate the magnitude of the RB effect, Fig. 2 shows the “accuracy difference” between the *identical* condition versus the *unrelated* condition, and between the *semantically related*/*synonymous* condition versus the *unrelated* condition, of this and all subsequent experiments).

**Table 1:** Mean accuracy in percentage in each experiment.

	Identical	Synonymous/Semantically related	Unrelated (Baseline)
*Picture*	^**†**^32 (5.08)	*30 (3.99)	44 (5.26)
***Word-General***			
One-character	*32 (6.20)	77 (3.90)	78 (3.47)
Two-character	*63 (4.53)	80 (3.99)	79 (3.67)
***Pictograph***			
Set I	*48 (6.91)	78 (5.14)	83 (4.15)
Set II	*42 (5.75)	54 (5.17)	64 (4.78)
***Pictograph-Sentence***			
Set I	*32 (3.97)	58 (5.08)	68 (5.13)
Set II	*46 (5.61)	87 (3.54)	84 (3.87)
***Pictograph -Replication***			
***Pictograph***			
Set I	*42 (4.58)	75 (2.44)	69 (1.78)
Set II	*23 (3.29)	60 (2.36)	64 (2.48)
***Pictograph-Sentence***			
Set I	*42 (2.73)	65 (2.36)	68 (2.81)
Set II	*50 (3.52)	81 (2.48)	85 (2.00)

^*^p < 0.05; ^†^*p* = 0.05. The tests of significance were based on logit mixed model with the accuracy in the *unrelated* condition used as the baseline. The numbers in the parentheses indicate one standard error of the mean across subjects.

### Word-General experiment

Based on picture RB, we used the same analysis method to test the semantic RB effect for Chinese words in the *Word-General* experiment (Fig. 1b,c), where the two critical words (C1 and C2) could be identical, synonymous or unrelated. Two major types of Chinese words, one-character words and two-character words were used; however, no semantic RB effect was manifested, as revealed by the lack of significant difference between the accuracy in the unrelated condition and that in the synonymous condition for one-character words (*z* = −0.56, *p* = 0.58) and for two-character words (*z* = −0.24, *p* = 0.81). Nevertheless, an orthographic RB effect was significant, as evidenced by the significantly lower accuracy in the identical condition than that in the unrelated condition for one-character words (*z* = −8.77, *p* < 0.001) and for two-character words (*z* = −4.34, *p* < 0.001).

### Pictograph experiment

Before we reached the conclusion that Chinese words are not processed like pictures, we tried several other conditions that were likely to induce a semantic RB effect. In the *Pictograph* experiment, only pictographs (a subset of Chinese words created by mimicking the physical shape of the indicated object) were used as critical items. Chinese synonyms that are two pictographs are rare, and we thus had to adopt a next best solution to this problem. We divided the experimental trials into two sets: In Set I, only the C1s in the synonymous condition were pictographs, and in Set II, C1 and C2 in each pair were both pictographs, but they were *semantically related* rather than completely *synonymous* (e.g.,  meaning “feather” and  meaning “bird”). However, we did not find significant RB effect for semantically related words. There was no significant accuracy difference between the synonymous (or semantically related) condition and the unrelated condition for Set I (*z* = −1.01, *p* = 0.31) and for Set II (*z* = −1.68, *p* = 0.09), whereas the accuracy in the identical condition was significantly lower than that in the unrelated condition for Set I (*z* = −5.31, *p* < 0.001) and Set II (*z* = −3.58, *p* < 0.001).

### Pictograph-Sentence experiment

In the *Pictograph-Sentence* experiment, Chinese pictographs were presented in sentences to provide rich semantic context for semantic RB to exert its effect (Fig. 1d). Similar to previous experiments, there was no significant accuracy difference between the synonymous (or semantically related) condition and the unrelated condition for Set I (*z* = −1.03, *p* = 0.30) and Set II (*z* = 0.76, *p* = 0.45), and the accuracy in the identical condition was significant lower than that in the unrelated condition for Set I (*z* = −4.07, *p* < 0.001) and Set II (*z* = −4.34, *p* < 0.001).

### Pictograph-Replication experiment

To confirm the reliability of the absence of semantic RB effect, we ran the *Pictograph-Replication* experiment with similar procedures to those in the Pictograph and Pictograph-Sentence experiments, but with more participants and more items for each participant, and even so no semantic RB was observed. There was no significant accuracy difference between the synonymous (or semantically related) condition and the unrelated condition for Pictograph/Set I (*z* = −0.49, *p* = 0.62), Pictograph/Set II (*z* = −0.48, *p* = 0.64), Pictograph-Sentence/Set I (*z* = −0.27, *p* = 0.79), and Pictograph-Sentence/Set II (*z* = −0.97, *p* = 0.33), but the accuracy in the identical condition was significantly lower than that in the unrelated condition for Pictograph/Set I (*z* = −3.17, *p* = 0.002), Pictograph/Set II (*z* = −4.5, *p* < 0.001), Pictograph-Sentence/Set I (*z* = −4.91, *p* < 0.001), and Pictograph-Sentence/Set II (*z* = −6.87, *p* < 0.001).

Throughout the study, orthographic or form RB effects were constantly found in all experiments, whereas the semantic RB effect was only significant in the experiment with picture materials but not with word materials. We thus conclude that the processing of Chinese words is more like that for English words than for pictures.

## Discussion

Our results can be explained by Bavelier’s^32^ model: Word recognition proceeds too rapidly for the individual tokenization of orthography to allow any room for semantics to exert any effect on RB. This is because once the type-token binding process has been completed, the semantic code does not have a chance to be individuated and consolidated into visual short-term memory. In contrast to words, picture identification is assumed to depend on extraction of meaning at a very early stage, and thus visually non-identical pictures have a chance to activate their meaning representations quickly enough to render RB if both carry the same or similar meanings. If this is true, the absence of RB for Chinese synonyms with different orthographies implies that they are individually tokenized before they are processed up to a semantic level, and hence, that their semantic information has no effect on RB.

The absence of RB for Chinese synonyms indicates similarities in processing between Chinese and English words (rather than pictures) in terms of the type-token binding process, even though the orthography-meaning relationship may be closer in Chinese than in English. In English, this relationship is generally arbitrary, except for morphemes (e.g., -er typically refers to a person) and other idiosyncratic exemplars (e.g., sn- sometimes carries the meaning related to nose; sneeze, sniffle, snout, snore). The mapping in English largely depends on the orthography-phonology relationship (e.g., the letter ‘d’ is almost always pronounced/d/). For Chinese words, the orthography-meaning mapping is more reliable than the orthography-phonology mapping.

The effects of the reliable orthography-meaning mapping for Chinese words are two-fold. From the perspective of Chinese words as visual stimuli, they convey reliable semantic cues so even non-Chinese users can still guess the meanings of Chinese words at an above-chance level^5–8^. Also, the reliable orthography-meaning mapping raises the likelihood of automatic semantic processing, so that the meanings of Chinese words exert their effects even for a task that does not require semantic processing such as color naming^10^. Compared to English words, Chinese words may elicit a higher degree of automatic semantic processing, but the orthographic codes are still activated prior to the semantic ones when the token is consolidated into short term memory, so semantic RB can be avoided, which is similar to English words instead of pictures. Even with the difference in the two writing systems, the processing of Chinese words is still more similar to English words than to pictures as revealed by the RB paradigm used here.

In the literature, there is a trend that for studies probing a relatively later time window (>300 ms), Chinese words are processed more like English words but not pictures^13,20,21^; for studies that probed a relative earlier time window, Chinese words are processed like pictures^18^. The present study probes a time window earlier than 100 ms, yet the results were consistent with the former case. Our speculation is that at a very early stage of information processing, the complex geological features both in Chinese words and in pictures might be the cause of their processing similarity. For example, Chinese words are constructed by strokes varying in shape, direction and length, which resemble the complex contour structure of an object. However, the contours of the Englsih alphabet are relatively simple. After the features have been combined into a word, Chinese word processing becomes more similar to that of English words. In the current study, although the presentation time was short, RB only occurred after features of C1 have been combined into a word. In this case, Chinese words are processed more like English words than pictures.

How are Chinese words processed? According to the *multi-level interactive-activation* model^40–42^, the orthographic, phonological and semantic representations are three necessary components for reading Chinese. The orthographic subsystem constitutes four hierarchical levels: strokes, radicals, characters, and words. The representations at the lower level send activation signals to the linked representations at the higher level, while the representations at the same level inhibit each other if they receive the same activation from the lower level. This model assumes that meaning can only be accessed through activation of character-level representation by connecting to their corresponding semantic representations through a concept unit called “lemma”. In the case of the current study, when synonyms are repeated twice, before the observer retrieves their meanings, they had been represented as two different “lemmas” and the tokenization process of one item did not interfere with the tokenization of the second item. This can explain the absence of RB for synonyms found here.

In recent years, studies have shown that semantic processing of words could be very efficient, even prior to the involvement of consciousness, for alphabetic words^43–46^, and for logographic words such as Chinese^47–49^. In the study of Yeh *et al*.^47^, for example, Chinese words were rendered invisible under visual crowding, where conscious identification of a salient object is impaired when it is surrounded by flankers. In that study, Chinese words used as primes were rendered unidentifiable due to crowding, but they still facilitated the recognition of the target that was presented subsequently. More importantly, this facilitation effect was found when the prime and the target were semantically related. This suggests that the semantic information of the prime had exerted its effect even when it was not consciously identified.

If Chinese words can activate their semantic representations without consciousness, how does it reconcile with the current study where Chinese words do not activate their semantic representations directly? Note that the involvement of consciousness and the time of processing can be dissociated^50,51^. An unconscious process might still require a relatively long time. Chinese words might activate their semantic representations without consciousness, but unconscious semantic activation might still occur later than unconscious orthographical activation, where synonyms can be separately tokenized so that RB can be avoided.

In conclusion, we found RB for semantically related pictures but not for Chinese synonyms, even for the most picture-like pictographs and when semantic cues were provided by embedding the critical items in sentences. Although this topic has been investigated and hotly debated^10,13,18,20,21^, we believe that the RB paradigm is a better method to tap into the early level of word processing, compared to previous studies that used a relative long presentation time. The absence of RB for Chinese synonyms indicates that Chinese words are not processed like pictures, even though they may have a closer orthography-meaning mapping relationship than that in English words. Furthermore, this work extends beyond the Western population, so we believe that the finings in this study enhance our understanding of the visual processing of symbolic materials in a more general human population (the WEIRD issue, see the study of Henrich, Heine & Norenzayan^52^).

## Methods

### Participants

All participants were undergraduates of National Taiwan University, approximately 18–22 years of age, and native Mandarin speakers. The numbers of participants were 21, 21, 30, 30, 33, and 59 in the Picture, Word-General/one-character, Word-General/two-character, Pictograph, Pictograph-Sentence, and Pictograph-Replication experiments, respectively. This study was approved by the ethics committee of the Department of Psychology at National Taiwan University, and all experiments were conducted in accordance with research subject guidelines and regulations laid down by the ethics committee. All participants gave informed consent prior to data collection.

### Stimuli and Design

See Appendices A to G for stimuli (pictures, words, and symbols) used.

#### Picture experiment

Stimuli were displayed on a 15-inch EIZO FlexScan F553M color monitor, with 54 cm viewing distance in a dimly lit chamber. Each trial consisted of an RSVP sequence of seven items, sandwiched by two displays that contained a fixation sign to signal the start and end of the sequence. These items (26° wide × 20° high) were presented at a rate of 100 ms/item in the following order: S, S, C1, IR, S, C2, and S (Fig. 1a).

C2s consisted of 27 pictures. Three C1s were then chosen in each of the three (*identical*, *semantically related*, and *unrelated*) conditions for any given C2. In order to control for picture complexity and familiarity, the C1s of the *semantically related* and *unrelated* conditions were chosen from Wang’s^53^ study, in which complexity and familiarity indices of 132 object contours were provided. The indices of complexity and familiarity were not significantly different between the C1s in the *semantically related* condition and the *unrelated* condition. Some C2s were chosen from Wang’s^53^ study, and some were drawn by the author SYL. The 27 C2s in each condition were divided into three parts (part I, II, III). Three versions of test trials were designed (version A, B, C), with each version containing three parts of 9 trials in each of the three conditions, and given to three groups of participants in a Latin-square design. For example, the test trials in version A comprised part I, II, and III of the *identical, semantically related, and unrelated* conditions, respectively; those in version B comprised part II, III, I, and version C part III, I, and II.

Each participant performed 27 test trials and 10 filler trials. The filler trials contained only two pictures not used in the test trials. The purpose of adding these filler trials was to provide RSVPs that contained two pictures, comparable to the situation in which RB occurs and the participants only perceive two pictures in a three-picture trial.

One may suspect that semantically related pictures are more likely to possess identical physical features, so the RB effect observed in this experiment might be caused by physical similarity rather than semantic similarity. To rule out this account, we quantified the physical similarity of the stimuli we used by means of the *normalized cross-correlation* technique, which is widely used in the application of image processing to identify a template or a pattern in a picture^54^ (See Appendix A for detailed descriptions).

#### Word-General experiment

For this and subsequent experiments, stimuli were displayed on a 21-inch calibrated EIZO FlexScan T966 color monitor (refresh rate = 70 Hz), and participants sat at a viewing distance of 60 cm in a dimly lit chamber.

Each trial contained an RSVP sequence (Fig. 1b,c), sandwiched by two displays that contained just a fixation cross (1.15° wide × 1.24° high). An RSVP contained 7 items presented at a rate of 57 ms/item in the following order: S, S, C1, IR, S, C2, and S. We used the 57 ms/item presentation rate based on our previous finding^55^ that this presentation duration could induce large RB for Chinese words. IR and C2 were in Chai font (, 1.15° × 1.24°), and C1 was in Hei font (, 1.53° × 1.43°). The words in Chai font resemble the words written in Chinese calligraphy. The strokes tend to have curvy features and generally differ in width; the words in Hei font have bolder strokes and the strokes are equal in width. C1 and C2 differed in both font and size to avoid visual masking^56^. The four Ss were selected randomly without replacement from a set of 33 symbols. For synonymous C1-C2 pairs, they did not share a common radical. Therefore, if any RB effect could be found between two synonymous Chinese words, it could not be explained by the physical similarity between the two words.

In both the one-character word and two-character word conditions, 27 words were chosen as C2s. Three different C1s were then chosen in each of the three conditions (*identical*, *synonymous*, and *unrelated* conditions) for any given C2. Analyses of the C1s in the three conditions showed no significant differences in word frequency^57,58^ or stroke count. The 27 C2s in each condition were divided into three parts (part I, II, III), arranged as a Latin-square design as in the Picture experiment. In the one-character word condition, each participant conducted 27 test trials (Appendix B) and 20 filler trials. The filler trials contained only two words, and C2 was replaced by a symbol. In the two-character word condition, an experimental session consisted of 39 trials, of which 27 were test trials (Appendix C), and 12 filler trials, in which C2 was replaced by two symbols.

#### Pictograph experiment

An experimental session consisted of 28 trials. Among them, 18 were test trials (half of them were from Set I, where only C1 were pictographic words; and half of them from Set II, where both C1 and C2 were pictographic words, but the *synonymous* condition was substituted by the *semantically related* condition. The words used in the experiment are shown in Appendices D and E). The other 10 trials were filler trials, in which C2 was replaced by a symbol. All other details were identical to the one-character word condition in the Word-General experiment. For synonymous or semantically related C1-C2 pairs, they did not share a common radical in either Set I or Set II.

#### Pictograph-Sentence experiment

Each trial contained one sentence with C1 and C2 embedded within it. A sentence consisted of eight to 11 words and two to five symbols, with the total number of items being constant at 13 for each trial. The lag between C1 and C2 was two to three words, and C1 and C2 had different fonts as previous experiments to avoid masking. Words in the first six frames (including C1) were in Hei font (, 1.53° × 1.43°), and those in the subsequent seven frames (including C2) were in Chai font (, 1.15° × 1.24°). The order of C1-C2 were identical to those in the Pictograph experiment except for the pair of  (“bamboo”) and  (“tree”), in which the order of the two words were swapped to form a sentence.

Each RSVP consisted of one sentence presented at the fixation location with one one-character word at a time at a rate of 98 ms per word. Kanwisher and Potter^33^ presented words in sentences with a speed of 117 ms per word, and Bavelier^32^ presented pictures and words in sentences at a rate of 83 ms per frame. We chose a presentation speed most appropriate for Chinese words as judged from pilot trials. The participant was requested to provide an oral report of the RSVP, as it was perceived, and to ignore grammar or logic.

An experimental session consisted of 18 test trials (Appendices F and G) and 10 filler trials. The sentences in the test trials were constructed to be grammatically correct. The 10 filler trials were either ungrammatical or illogical, to discourage guessing for the purpose of reconstructing grammatically correct sentences.

#### Pictograph-Replication experiment

Instead of a Latin-square design, a completely within-subject design was used in this experiment. Each participant was shown 168 trials comprised of all the possible stimuli used in the Pictograph experiment and the Pictograph-Sentence experiment.

### Procedure

#### Training stage (the Picture experiment only)

All the pictures, including the stimuli of the test trials and filler trials, and the name of each picture shown below it were presented one after another. The participant was required to memorize the name of each picture and use the names to indicate what they saw in the test stage as much as possible. Each picture, together with the name, was shown for five seconds, and if they thought they had memorized the name for a shorter duration, they could press the space bar to see the next picture.

#### Test Stage (all experiments)

The participant initiated a trial by pressing the space bar. A tone sounded for 150 ms to signal the start of a trial, followed by a fixation cross for one second. Then the RSVP sequence was shown, after which the fixation sign reappeared until the participant responded by speaking out (experiments where critical items were embedded in sentences) or writing down the names (experiments where critical items were embedded in lists) for the pictures or words they had seen. No feedback was given. Spoken responses were recorded on a digital sound recorder.

Prior to the experimental session was the practice session, which contained 10 trials. The procedure of the practice session was the same as that in the experimental session, but the stimuli used were exclusively different from those used in the experimental session.

#### Rating (all experiments with words as critical stimuli)

After completing the experimental session, each participant was given a questionnaire, where the C1-C2 word pairs used in the *synonymous* (or semantically related) and *unrelated* conditions were printed. They were to rate the semantic relationship between each C1-C2 pair on a scale from 1 (unrelated) to 6 (semantically identical), to ensure that the synonyms used in the experiment were perceived to be synonymous for the participants. The rating results are shown in Appendix H. All the C1-C2 pairs were perceived to be significantly more semantically related in the synonymous condition (or semantically related condition) than in the unrelated condition.

For experiments with pictographs as critical stimuli, participants were additionally asked to judge whether the individual words looked like pictures, with a simple yes-no response for each word. The pictographs were perceived to be significantly more like pictures than non-pictographs, as demonstrated by the substantial higher proportion of the “yes” responses for the pictographic words than for the general Chinese words. Please see the detailed statistical analysis in Appendix H.

### Data analysis

The accuracy data were analyzed using logit mixed model^39^ in R. In all experiments, the fixed effect factor was Relatedness (*identical, semantically related*, or *unrelated*), and the random effect factors were Subject and Item. In the Word-General experiment, we additionally used Number of Character as a fixed effect factor. In experiments where pictographs were used as critical stimuli, Set (Set I versus II) was added as a fixed effect factor. In all experiments, the modeling results based on the full set of parameters for random effects (the random *intercepts* for Subject and Item, and their random *slopes* for the effect of Relatedness) showed high correlations between the random intercept of Subject and further random effects of Subject (*rs* > 0.9 or *rs* < −0.9), implying possible over-parameterization. Therefore, the random slope of Subject was removed in the final models reported. The Pictographs experiment, in particular, also showed high correlations between the random intercept of Item and some further Item random effects, and thus the random slope of Item was removed.

## Electronic supplementary material

Appendices
