# Supplementary material for: Does “a picture is worth 1000 words” apply to iconic Chinese words? Relationship of Chinese words and pictures
Source: Sci Rep. 2018 May 29;8:8289. doi: 10.1038/s41598-018-25885-9 (PMC5974396; doi:10.1038/s41598-018-25885-9)
Supplement: Supplementary file 1 — Appendices [file 41598_2018_25885_MOESM1_ESM.pdf]

Supplemental Materials for:

Does "a picture is worth 1000 words" apply to iconic Chinese words? Relationship of Chinese words and pictures

Shih-Yu Lo[1,2] and Su-Ling Yeh[3,4,5,6*]

[1]Institute of Communication Studies, National Chiao Tung University, Hsinchu, Taiwan

[2]Center for General Education, National Chiao Tung University, Hsinchu, Taiwan

[3]Department of Psychology, National Taiwan University, Taipei, Taiwan

[4]Graduate Institute of Brain and Mind Sciences, National Taiwan University, Taipei, Taiwan

[5]Neurobiology and Cognitive Science Center, National Taiwan University, Taipei, Taiwan

[6]Center for Artificial Intelligence and Advanced Robotics, National Taiwan University, Taipei, Taiwan

*Corresponding Author:
Su-Ling Yeh
Department of Psychology
National Taiwan University
Taipei 10617, Taiwan
Phone: (886) 2-33663097
Fax: (886) 2-23629909
Email: suling@ntu.edu.tw

Appendix A: Stimuli used in the Picture experiment

Identical	*Semantucally related*	*Unrelated*	
C1	C1	C1	C2
Name of the picture	Name of the picture	Name of the picture	Name of the picture
Desk Lamp	Bed Lamp	Whistle	Desk Lamp
Candle	Flashlight	Skirt	Candle
Watch	Clock	Tent	Watch
Chair	Sofa	Scissors	Chair
Helicopter	Airplane	Rabbit	Helicopter
Car	Bus	Compass	Car
Piano	Grand Piano	Umbrella	Piano
Hunting Gun	Handgun	Glasses	Hunting Gun
Fan	Electric Fan	Elephant	Fan
Cell Phone	Telephone	Fish	Cell Phone
Lotus	Periwinkle	Traffic Light	Lotus
Microscope	Binoculars	Guitar	Microscope
Weighing Scale	Platform Scale	Electric Pot	Scale
Ship	Sailboat	Fork	Ship
Christmas Hat	Western Hat	Refrigerator	Christmas Hat
High Heel	Slipper	Spoon	High Heel
Ice Cream	Popsicle	Pipe	Ice Cream
Pot	Frying Pan	Bat	Pot
Knife	Axe	Hair Dryer	Knife
Sword	Bow and Arrow	Camel	Sword
French Horn	Trumpet	Iron	French Horn
Door Knob	Lock	Bucket	Door Knob
Inline Skate	Roller Skate	Tank	In-line Skate
Train	Metro	Ostrich	Train
Swallowtail	White Butterfly	Mailbox	Swallowtail
Shrimp	Crab	Audio	Shrimp
Razor	Electric Razor	Thermos	Razor
Mean picture complexity (measured by the number of geons)			
	8.9	8.8	
Mean familarity (measured by the rating results from 41 observers on a five-point scale)			
	4.1	4.3	

Note: 1. There was no difference between the picture complexity ($t(26) = 0.15, p > .1$) of the *semantically-related,* and unrelated conditions. The same is true for their picture familarity ($t(26) = 0.88, p > .1$).

2. All the C1s in the semantically related condition and the unrelated condition were chosen from the database established by Wang[53], who also provides indices for picture complexity and familiarity.

3. Some C2s (also corresponding C1s in the identical condition) were chosen from the database established by Wang[53], and the others were drawn by the author SYL.

4. A written permission to use the object contour pictures from Wang's study has been granted by Chinese Journal of Psychology.

Continue on next page

Normalized cross-correlation analysis for the Picture experiment

All the C1s and C2s were first converted into two-dimensional matrices of gray-scale values. A normalized cross-correlation coefficient matrix is expressed as:

$$Corr(x,y) = \frac{\sum_{x',y'}[C1(x',y') - \overline{C1}][C2(x+x',y+y') - \overline{C2}]}{\sqrt{\sum_{x',y'}[C1(x',y') - \overline{C1}]^2 \sum_{x',y'}[C2(x+x',y+y') - \overline{C2}]^2}}$$

, where C1 and C2 are two *M* x *N* matrices, and $\overline{C1}$ and $\overline{C2}$ indicate their mean values. The x' and y' refer to the positions in the picture C1 or C2, and x and y refer to the displacement of C1 and C2. For example, Corr (0,0) refers to the image correlation when C1 and C2 are exactly overlapping; Corr (2, 3) refers to the images correlation when C2 is placed two pixels horizontally and three pixels vertically shifted from C1. The *degree of similarity* is represented by the peak value of this normalized cross-correlation coefficient matrix. In other words, if two pictures are exactly the same, the peak value should appear at Corr (0,0). But in some cases, the same object might be located on the right side in C1 but on the left side in C2, and the peak value would appear somewhere other than Corr (0,0). The statistical analysis showed that the mean *degree of similarity* for the pictures in the *semantically related* condition (0.08) was not significantly higher ($t(26) = 0.55$, $p = 0.59$) than that in the *unrelated* condition (0.078). Thus, it is very unlikely that the accuracy difference between the *semantically related* condition and the *unrelated* condition was due to their physical similarity.

Appendix B: Stimuli used in the Word-General experiment / one-character condition

	Identical		*Synonymous*		*Unrelated*			
	C1		C1		C1		C2	
	Word	meaning	Word	meaning	Word	meaning	Word	meaning
	冠	Hat	帽	Hat	溪	Stream	冠	Hat
	幼	Child	孩	Child	段	Segment	幼	Child
	罩	Cover	蓋	Cover	察	To Discern	罩	Cover
	足	Foot	腳	Foot	樹	Tree	足	Foot
	卵	Egg	蛋	Egg	季	Season	卵	Egg
	冊	Book	書	Book	喜	Happy	冊	Book
	凳	Chair	椅	Chair	祕	Secret	凳	Chair
	臥	To Lie	躺	To Lie	瞬	Fast	臥	To Lie
	傾	Tilted	歪	Tilted	昆	Brother	傾	Tilted
	碟	Dish	盤	Dish	雪	Snow	碟	Dish
	頸	Neck	脖	Neck	煤	Coal	頸	Neck
	母	Mother	娘	Mother	鼓	Drum	母	Mother
	日	Sun	陽	Sun	停	To Stop	日	Sun
	履	Shoe	鞋	Shoe	湯	Soup	履	Shoe
	罐	Bottle	瓶	Bottle	階	Ladder	罐	Bottle
	驟	Fast	急	Fast	吳	(a family name)	驟	Fast
	扔	Throw	丟	Throw	朱	Red	扔	Throw
	疏	Rare	少	Rare	及	And	疏	Rare
	圈	Circle	環	Circle	斯	This	圈	Circle
	房	House	室	House	笑	Laugh	房	House
	犬	Dog	狗	Dog	詞	Word	犬	Dog
	壤	Soil	泥	Soil	弦	String	壤	Soil
	兵	Soldier	卒	Soldier	余	I	兵	Soldier
	面	Face	臉	Face	銘	To Engrave	面	Face
	乳	Milk	奶	Milk	役	Labor	乳	Milk
	沒	No	無	No	些	Some	沒	No
	食	Food	吃	Food	組	Group	食	Food
Mean word frequency	563		519		482		563	
Mean stroke count	10		11		11		10	

Note: 1. There was no difference between the word frequencies (*F(2, 52) = 0.13, p > .1)* of the *identical, synonymous,* and *unrelated conditions.* The same is true for their stroke counts (*F(2, 52) = 0.02, p > .1*).

2. Word (one character) frequency is based on Tsai[57] and is expressed as number of occurrences per 1.72 million.

3. The 33 symbols used were: & ∗ ∮ ◎ § @ ♂ $ ΦΩ≠≧≒∞ ¢ %ⅦΣ︶︵☆◇▽△⊿★▲◆◤◢◣◥●

Appendix C: Stimuli used in the Word-General experiment / two-character condition

	Identical		*Synonymous*		*Unrelated*			
	C1		C1		C1		C2	
	Word	meaning	Word	meaning	Word	meaning	Word	meaning
	老公	Husband	丈夫	Husband	疫苗	Vaccine	老公	Husband
	蕃薯	Sweet Potato	地瓜	Sweet Potato	四肢	Limb	蕃薯	Sweet Potato
	廁所	Toilet	茅房	Toilet	請帖	Invitation	廁所	Toilet
	奶媽	Nanny	保母	Nanny	大海	Sea	奶媽	Nanny
	元首	Leader	領袖	Leader	機械	Machine	元首	Leader
	僧侶	Monk	和尚	Monk	汽油	Gasoline	僧侶	Monk
	照相	Photography	攝影	Photography	治療	Cure	照相	Photography
	口吃	Stammer	結巴	Stammer	虛胖	Obese	口吃	Stammer
	憤怒	Angry	生氣	Angry	聰明	Smart	憤怒	Angry
	客運	Bus	巴士	Bus	幼兒	Child	客運	Bus
	旅社	Hotel	賓館	Hotel	瓷器	Procelain	旅社	Hotel
	寢室	Room	臥房	Room	句點	Dot	寢室	Room
	搭檔	Partner	夥伴	Partner	貨物	merchandise	搭檔	Partner
	鐵鎚	Hammer	榔頭	Hammer	稀飯	Rice	鐵鎚	Hammer
	開刀	Surgery	手術	Surgery	留言	Message	開刀	Surgery
	授課	To Teach	教學	To Teach	購買	To Purchase	授課	To Teach
	簡單	Easy	容易	Easy	合作	Cooperation	簡單	Easy
	漂亮	Beautiful	美麗	Beautiful	快速	Fast	漂亮	Beautiful
	黃昏	Dusk	傍晚	Dusk	香菇	Mushroom	黃昏	Dusk
	夾克	Jacket	外套	Jacket	河川	River	夾克	Jacket
	知識	Knowledge	學問	Knowledge	泡麵	Noodle	知識	Knowledge
	宦官	Eunuch	太監	Eunuch	瓦斯	Gas	宦官	Eunuch
	歲數	Age	年齡	Age	網頁	Webpage	歲數	Age
	調羹	Spoon	湯匙	Spoon	掌聲	Clapping	調羹	Spoon
	痊癒	To Recover	康復	To Recover	訓話	Discipline	痊癒	To Recover
	注射	Injection	打針	Injection	爬山	Climbing	注射	Injection
	有錢	Rich	富裕	Rich	精準	Precise	有錢	Rich
Mean word frequency	27		54		47		27	
Mean stroke count	22		20		20		22	

Note: 1. There was no difference between the word frequencies (*F(2, 52) =2.06, p > .1)* of the *identical, synonymous,* and *unrelated conditions.* The same is true for their stroke counts (*F(2, 52) = 1.14, p > .1*).

2. Word (two characters) frequency is based on *Statistical Reports of Chinese Character and Word Frequency of Chinese Dictionary, Brief Version,* published by Ministry of Education of Taiwan[58], and is expressed as number of occurrences in a pool of 530,452 words.

Appendix D: Stimuli used in the Pictograph experiment / Set I

	Identical		*Synonymous*		*Unrelated*			
	C1		C1		C1		C2	
	Word	meaning	Word	meaning	Word	meaning	Word	meaning
	狗	Dog	犬	Dog	几	Table	狗	Dog
	窩	Nest	巢	Nest	卷	Paper	窩	Nest
	溪	Stream	川	Stream	戶	Window	溪	Stream
	陽	Sun	日	Sun	女	Female	陽	Sun
	娘	Mother	母	Mother	典	Classics	娘	Mother
	腳	Foot	足	Foot	史	History	腳	Foot
	液	Water	水	Water	光	Light	液	Water
	蛋	Egg	卵	Egg	巾	Towel	蛋	Egg
	書	Book	冊	Book	玉	Jade	書	Book
Mean word frequency	387		646		560		387	
Mean stroke count	11		6		5		11	

Note: 1. There was no difference between the character frequencies (*F (1, 8) = 2.93, p > .1)* of the *synonymous* and *unrelated conditions.* The same is true for their stroke counts (*F (1, 8) = 0.73, p > .1*).

2. Word (one character) frequency is based on Tsai [57], and is expressed as number of occurrences per 1.72 million.

Appendix E: Stimuli used in the Pictograph experiment / Set II

	Identical		*Semantically related*		*Unrelated*			
	C1		C1		C1		C2	
	Word	meaning	Word	meaning	Word	meaning	Word	meaning
	土	Soil	田	Field	江	River	土	Soil
	貝	Shellfish	魚	Fish	帝	Emperor	貝	Shellfish
	鳥	Bird	羽	Feather	灰	Gray	鳥	Bird
	竹	Bamboo	木	Tree	空	Empty	竹	Bamboo
	石	Stone	山	Mountain	王	King	石	Stone
	馬	Horse	羊	Sheep	岸	Shore	馬	Horse
	人	Human	子	Child	年	Year	人	Human
	耳	Ear	手	Hand	力	Force	耳	Ear
	矛	Lance	弓	Bow	穴	Cave	矛	Lance
Mean word frequency	2165		1085		1024		2165	
Mean stroke count	6		5		6		6	

Note: 1. There was no difference between the character frequencies ($F\ (1, 8) = 0.14,\ p > .1$) of the *synonymous* and *unrelated conditions.* The same is true for their stroke counts ($F\ (1, 8) = 2.12, p > .1$).

2. Word (one character) frequency is based on Tsai[57], and is expressed as number of occurrences per 1.72 million.

Appendix F: Stimuli used in the Pictograph-Sentence experiment / Set I

		Identical			*Synonymous*			*Unrelated*		
C2		C1			C1			C1		
狗	Dog	狗	Dog	這些土*狗*和貴賓*狗*是我的	犬	Dog	這些惡*犬*和貴賓*狗*是我的	几	Table	這些茶*几*和貴賓*狗*是我的
				These native dogs and poodles are mine.			These fierce dogs and poodles are mine.			These tables and poodles are mine.
窩	Nest	窩	Nest	花園裡蟲*窩*和燕*窩*遍佈	巢	Nest	森林裡鳥*巢*和燕*窩*遍佈	卷	Paper	櫃子上考*卷*和燕*窩*遍佈
				Insect nests and swallow nests are everywhere in the garden.			Bird nests and swallow nests are everywhere in the forest.			Examination sheets and swallow nests are everywhere on the closet.
溪	Stream	溪	Stream	那裡的大*溪*和小*溪*很有名	川	Stream	那裡的山*川*和小*溪*很有名	戶	Window	那裡的住*戶*和小*溪*很有名
				The big stream and the small stream over there are famous.			The mountains and the small stream over there are famous.			The residents and the small stream over there are famous.
陽	Sun	陽	Sun	太*陽*下撐*陽*傘可避暑	日	Sun	烈*日*下撐*陽*傘可避暑	女	Female	少*女*以撐*陽*傘來避暑
				Holding an umbrella can avoid the heat in the sun.			Holding an umbrella can avoid the heat in the sun.			The girl is holding an umbrella to avoid the heat in the sun.
娘	Mother	娘	Mother	你的*娘*親是我*娘*的學生	母	Mother	你的*母*親是我*娘*的同事	典	Classics	那部*典籍*是我*娘*的物品
				Your mother is my student.			Your mother is my colleague.			That classic book belongs to my mother.
腳	Foot	腳	Foot	他愛揉*腳*趾和用*腳*寫字	足	Foot	他愛踢*足*球和用*腳*寫字	史	History	他愛讀*史*書和用*腳*寫字
				He loves to rub the toes and write with toes.			He loves to play soccer and write with toes.			He loves to read history books and write with toes.
液	Water	液	Water	加了碘*液*使得血*液*變色	水	Water	加了開*水*使得血*液*變色	光	Light	透了銀*光*使得血*液*變色
				The iodine liquid changed the color of the blood.			The boiled water changed the color of the blood.			The silver light changed the color of the blood.
蛋	Egg	蛋	Egg	有鴨*蛋*和雞*蛋*在花園裡	卵	Egg	有龜*卵*和雞*蛋*在花園裡	巾	Towel	有毛*巾*和雞*蛋*在花園裡
				There are some duck eggs and chicken eggs in the garden.			There are some turtle eggs and chicken eggs in the garden.			There are some towels and chicken eggs in the garden.
書	Book	書	Book	他訂了古*書*和童*書*了吧	冊	Book	他訂了手*冊*和童*書*了吧	玉	Jade	他訂了愛*玉*和童*書*了吧
				He must have ordered ancient books and children's books.			He must have ordered dictionaries and children's books.			He must have ordered jellies and children's books.
Mean word frequency		387			646			560		
Mean stroke count		11			6			5		

Note: 1. There was no difference between the character frequencies ($F(1, 8) = 2.93$, $p > .1$) of the *synonymous* and *unrelated conditions.* The same is true for their stroke counts ($F(1, 8) = 0.73$, $p > .1$).

2. Word (one character) frequency is based on Tsai[57], and is expressed as number of occurrences per 1.72 million.

Appendix G: Stimuli used in the Pictograph-Sentence experiment / Set II

C2		Identical C1			Semantically related C1			Unrelated C1		
土	Soil	土	Soil	黃土邊的土質很肥沃	田	Field	稻田邊的土質很肥沃	江	River	長江邊的土質很肥沃
				The soil near the yellow soil is fertile.			The soil near the rice field is fertile.			The soil near Long River is fertile.
貝	Shellfish	貝	Shellfish	傳說的寶貝靠著貝類維生	魚	Fish	海邊的大魚靠著貝類維生	帝	Emperor	劇中的皇帝靠著貝類維生
				The legendary creature feeds on shells.			The big fish in the ocean feeds on shells.			The emperor in the drama feeds on shells.
鳥	Bird	鳥	Bird	許多鳥糞覆蓋在鳥身上	羽	Feather	許多羽毛覆蓋在鳥身上	灰	Gray	許多灰塵覆蓋在鳥身上
				Bird droppings are covering the birds.			Feathers are covering the birds.			Dust is covering the birds.
木	Tree	木	Tree	他拿著木笛以及木頭	竹	Bamboo	他扛著竹竿以及木頭	空	Empty	他捧著空罐以及木頭
				He is holding a wooden flute and a wooden stick.			He is carrying a bamboo stick and a wooden stick.			He is holding an empy bottle and a wooden stick.
石	Stone	石	Stone	這座石城佈滿石頭	山	Mountain	這座山上佈滿石頭	王	King	這座王國佈滿石頭
				This stone city is covered with stones.			This mountain is covered with stones.			This kingdom is covered with stones.
馬	Horse	馬	Horse	在木馬旁有匹馬在叫	羊	Sheep	一群羊中有匹馬在叫	岸	Shore	黃河岸上有匹馬在叫
				The horse near the wooden house whinnies.			The horse among the sheeps whinnies.			The horse on the bank of Yellow River whinnies.
人	Human	人	Human	人道是古人的美德	子	Child	子女是古人的財產	年	Year	年齡是古人的身分
				Humanity is the virtue of ancient people.			Children are assets of ancient people.			Age represents the status of ancient people.
耳	Ear	耳	Ear	他用假耳摀住耳朵	手	Hand	他用雙手摀住耳朵	力	Force	他用全力摀住耳朵
				He covers the ears with fake ears.			He covers the ears with hands.			He covers the ears with all his energy.
矛	Lance	矛	Lance	他發現短矛和長矛的遺跡	弓	Bow	他發現彈弓和長矛的遺跡	穴	Cave	他發現洞穴和長矛的遺跡
				He found historical ramains of short lances and long lances.			He found historical remains of slingshots and long lances.			He found historical remains of caves and long lances.
Mean word frequency		2102			1147			1024		
Mean stroke count		6			5			6		

Note: 1. There was no difference between the character frequencies ($F(1, 8) = 0.86$, $p > .1$) of the *synonymous* and *unrelated conditions.* The same is true for their stroke counts ($F(1, 8) = 1.70$, $p > .1$).

2. Word (one character) frequency is based on Tsai[57], and is expressed as number of occurrences per 1.72 million.

Appendix H: Rating results of C1-C2 semantic relationships and word iconicity

Part I: C1-C2 semantic relationship rating

The average rating values for the C1-C2 pairs in the *synonymous/semantically related* condition and for the *unrelated* condition were 5.04 versus 1.28 ($t(52) = 34.78$, $p<.0001$; *Cohen's d* = 9.65), 4.89 versus 1.16 ($t(52)=42.88$, $p<.0001$; *Cohen's d*=11.89), 4.99 versus 1.68 ($t(16)=13.70$, $p<.0001$; *Cohen's d*=6.85), 3.59 versus 2.11 ($t(16)=7.44$, $p<.0001$; *Cohen's d*=3.72), 5.32 versus 1.53 ($t(16)= 22.61$, $p<.0001$; *Cohen's d* = 11.31), 3.55 versus 1.76 ($t(16)=10.86$, $p<.0001$; *Cohen's d*=5.43), 4.84, versus 1.60 ($t(16)=18.65$, $p<.0001$; *Cohen's d* =9.33), and 3.30 versus 1.93 ($t(16)=9.72$, $p<.0001$; *Cohen's d* =4.86) respectively in the Word-General experiment / one-character condition, Word-General experiment / two-character condition, Pictograph experiment / Set I, Pictograph experiment / Set II, Pictograph-Sentence / Set I, Pictograph-Sentence / Set II, the Pictograph-Replication / Set I, and Pictograph-Replication / Set II.

Part II: C1-C2 iconicity rating (experiments with pictographs as critical stimuli)

The pictographic words were C1s in the *synonymous* condition in Set I, and both C1s and C2s in the *semantically related* condition in Set II. As to the rest of the words used in this experiment, we did not choose them based on whether they were pictographic or not, so their rating results were taken as an index of the baseline. The average proportions of the "yes" responses for the pictographic words and the baseline were 80% versus 34% ($t(52)=7.78$, $p<.0001$; *Cohen's d* = 2.16), 78% versus 33% ($t(52)=8.0$, $p<.0001$; *Cohen's d* = 2.22), and 73% versus 33% ($t(52)=9.61$, $p<.0001$; *Cohen's d*=2.67) respectively in the Pictograph, Pictograph-Sentence, and Pictograph-Replication experiments.
